# Supplementary material for: A BaSiC tool for background and shading correction of optical microscopy images
Source: Nat Commun. 2017 Jun 8;8:14836. doi: 10.1038/ncomms14836 (PMC5472168; doi:10.1038/ncomms14836)
Supplement: Supplementary Information — Supplementary Figures, Supplementary Notes and Supplementary References [file ncomms14836-s1.pdf]

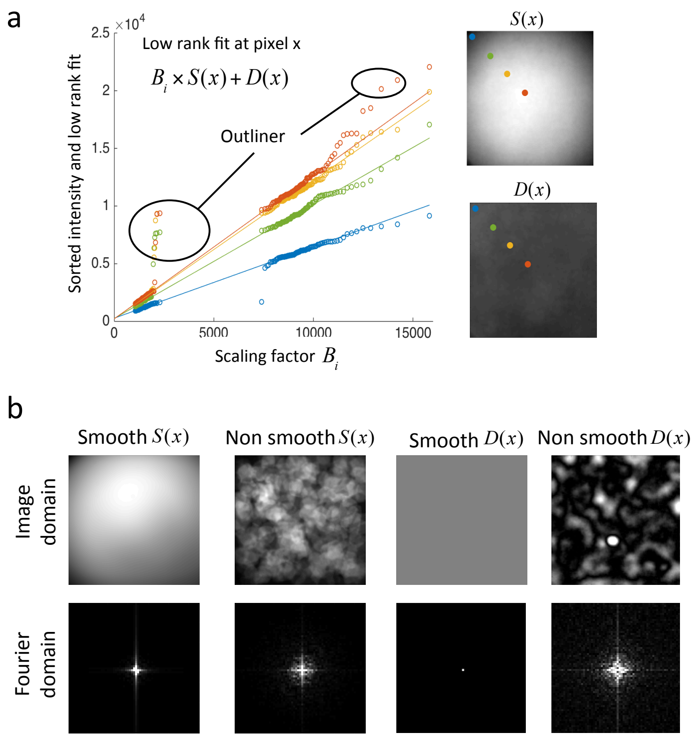

Supplementary Fig. 1: Sparse formulation of the objective function. (a) Sparse regularisation of the residue makes the low rank fitting robust to outliers. Each fitting line has a slope of S and a zero-crossing of D (refers to one colored pixel on the right). (b) Sparse constraints are imposed on the Fourier transformed S and D to enforce their smoothness in the image domain.

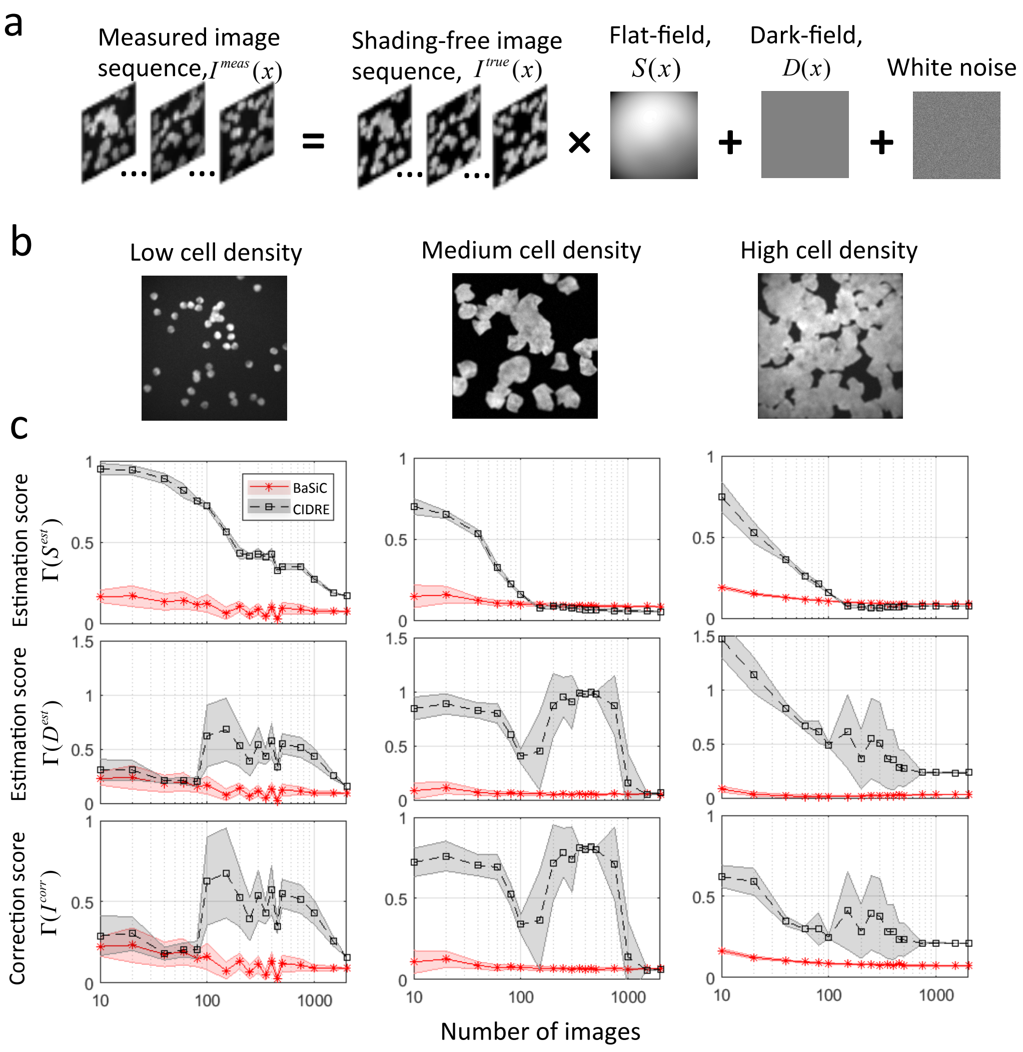

Supplementary Fig. 2: BaSiC needs a small number of images (<10) to achieve an accurate shading estimation and correction on synthetic images. (a) The measured image sequence, Imeas, is simulated by corrupting the shading-free image sequence, I^{true}, with a common flat-field S, dark-field D and white noise. (b) Three exemplary images of simulated image sequences of different cell confluencies (low: 7.3%, medium: 35.8%, high: 78.6%). (c) Error of the estimated S^{est} (top row), D^{est} (middle row) and the corrected images I^{corr} (bottom row), quantified by an estimation/correction score, measuring the mean absolute deviation (MAD) between the estimation/correction and the ground-truth, normalised by the MAD between a baseline estimation and the ground-truth. The baselines for S^{est}, D^{est} and I^{corr} are a uniform unit image ($S^{est}(x) = 1$), a uniform zero image ($D^{est}(x) = 0$), and the uncorrected image sequence I^{meas}, respectively. For each fixed number of images, n, we randomly selected 20 image subsets, made one estimation and correction on each subset. The solid line is the mean of the 20 subsets and the shaded area represents the standard deviation. In all three cell confluencies, BaSiC requires only a small number of images to accurately estimate S and D, whilst the competitive method, CIDRE, needs a much larger number of images to achieve the same accuracy. The discontinuity of CIDRE estimation on the dark-field D is due to a sudden change of smoothness regularisation in its default parameter setting at 100 images; in comparison, BaSiC chooses parameters that adapt to different image content and is able to maintain the accuracy.

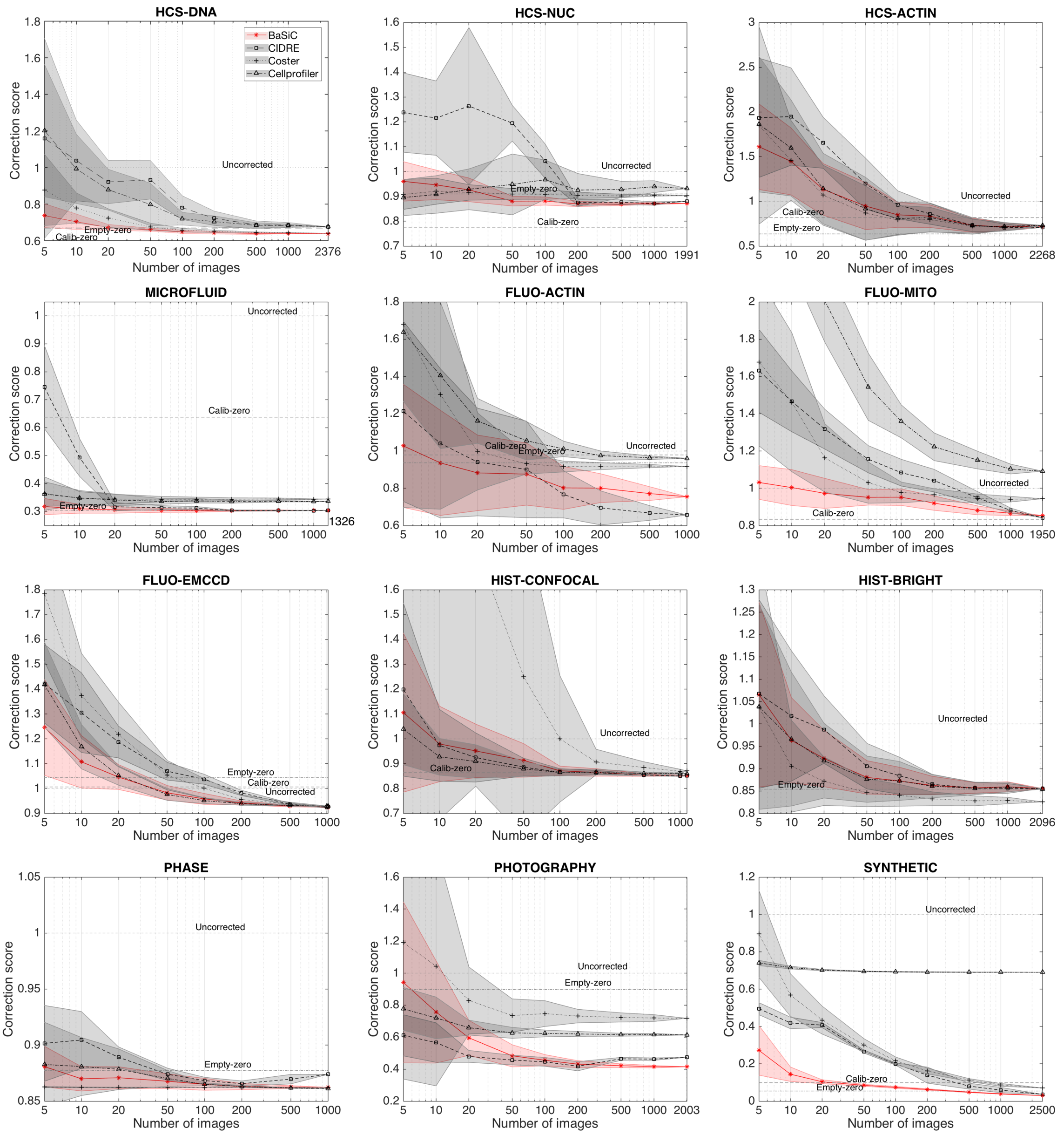

Supplementary Fig. 3: Correction score, $\Gamma'(I^{corr})$, as a function of the number of images used to train models of four retrospective methods (BaSiC, CIDRE, Coster and Cellprofiler) for 12 microscopy image collections. For each number of images, we trained 40 models on randomly selected subsets and evaluated the models on a test set of images with overlapping regions (see Smith et al. [6] for details). Each curve is the average score of the 40 subsets for each retrospective method. Shaded area represents the standard deviation. Baseline $\Gamma'(I^{corr}) = 1$ implies equivalent disagreement to the uncorrected image pairs, indicated by grey dotted lines. Scores for two prospective methods Calib-zero and Empty-zero are indicated by grey dashed and dot-dashed lines respectively. In all datasets, BaSiC's accuracy increases as the number of images increases, with 10-50 images to match the baseline score and 20-200 to match the prospective methods. In comparison to other retrospective methods, BaSiC mostly requires less images to reach a higher accuracy, particularly in datasets whereas the image background contains rich shading information (HCS-DNA, HCS-NUC, MICROFLUID, FLUO-MITO, FLUO-EMCCD, PHASE, SYNTHETIC).

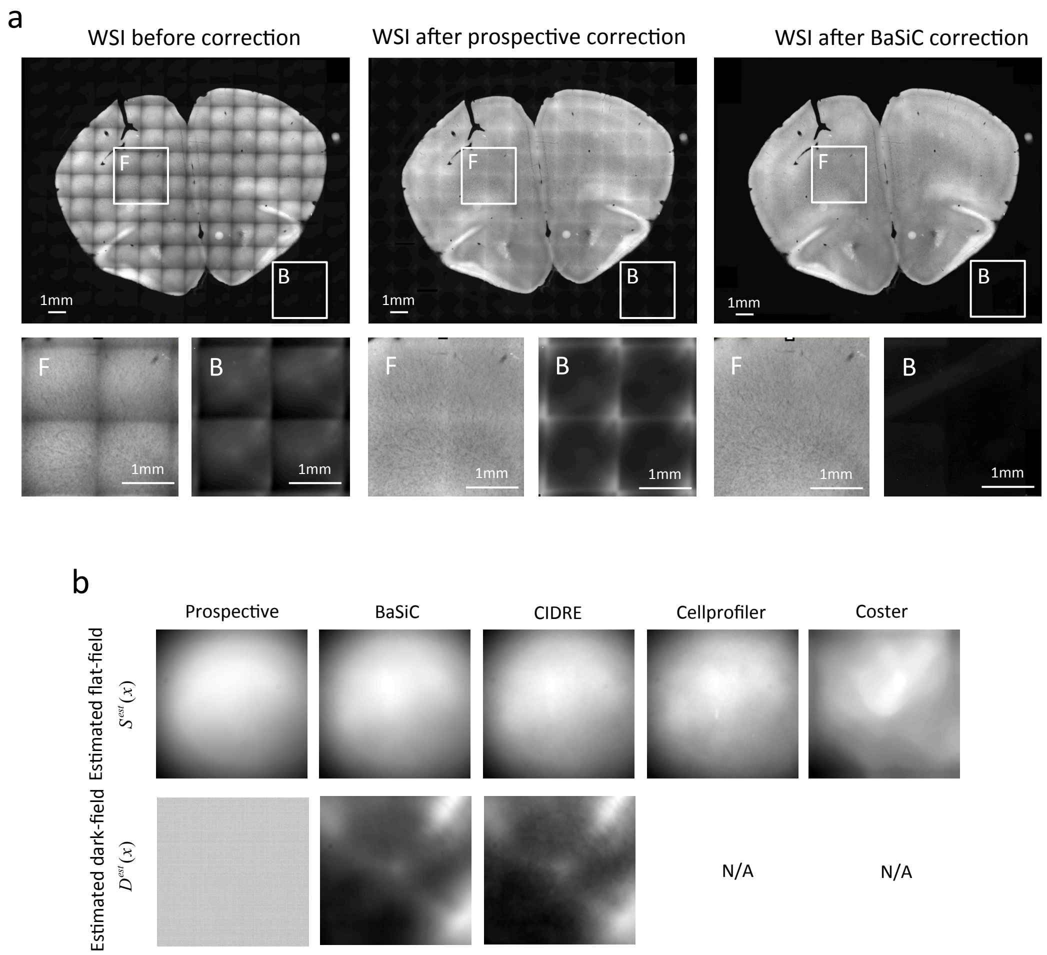

Supplementary Fig. 4: BaSiC is able to remove an additive artefact of stray light and the residual of excitation light entering in the camera due to imperfect filtering. By contrast, a prospective correction is not sufficient in this case because a dark-field calibration cannot capture the additive light, which is only present when the light source of a microscopy is on. (a) WSI of a mouse brain before any correction (left) shows a different shading pattern in the foreground vs the background. WSI after a prospective correction (middle) shows some residual checkerboard shading pattern, particularly in the background. In WSI after BaSiC correction (right), the checkerboard pattern disappears completely in both the foreground and background. (b) Estimated S and D of different methods. Whilst prospective, BaSiC, CIDRE and Cellprofiler can all make an accurate estimation of S, only BaSiC and CIDRE can estimate D correctly.

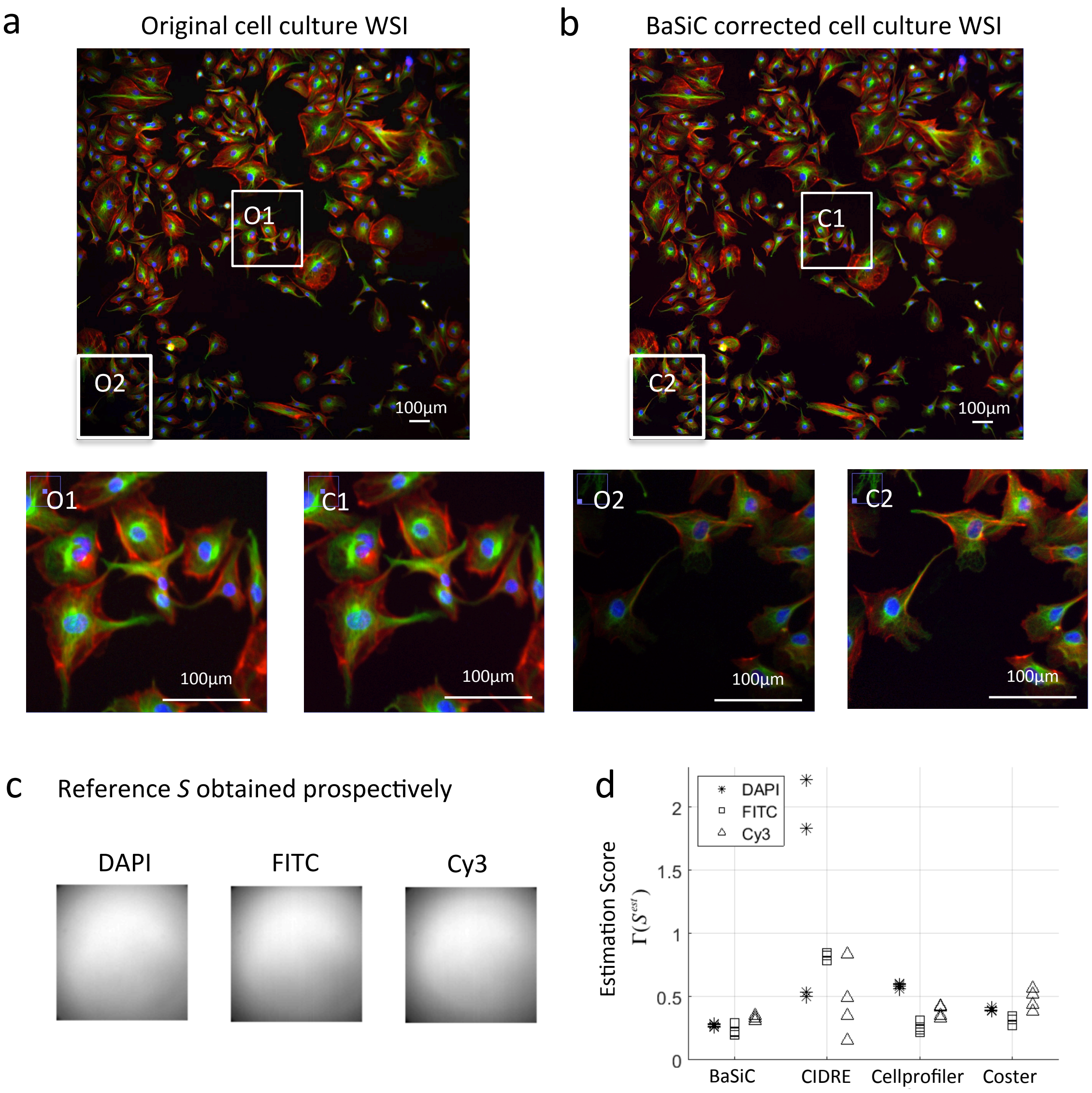

Supplementary Fig. 5: Four cell culture WSIs were collected using three fluorescence channels, DAPI, FITC and Cy3. For each retrospective method, one estimation was made for each WSI on each channel. Besides, reference dark-field and flat-field were obtained prospectively using a concentrated dye solution. (a) An exemplary original image tile. Cells at the edge of the image (O2) are much darker than cells at the centre of the image (O1). (b) The same image tile after BaSiC correction. With intensity correction, cells at the edge (C2) becomes much brighter. (c) Flat-field S obtained prospectively for all three channels using concentrated dye solution. (d) Estimation score, $\Gamma(S^{est})$, between the estimated S using different retrospective methods and the reference S obtained prospectively. A $\Gamma(S^{est}) = 1$ implies an equivalent error to the baseline error between reference S and a uniform image (no correction). The two outliers of CIDRE in the DAPI channel are due to image artefacts on these specimens (refer to Fig. 2c-e for details).

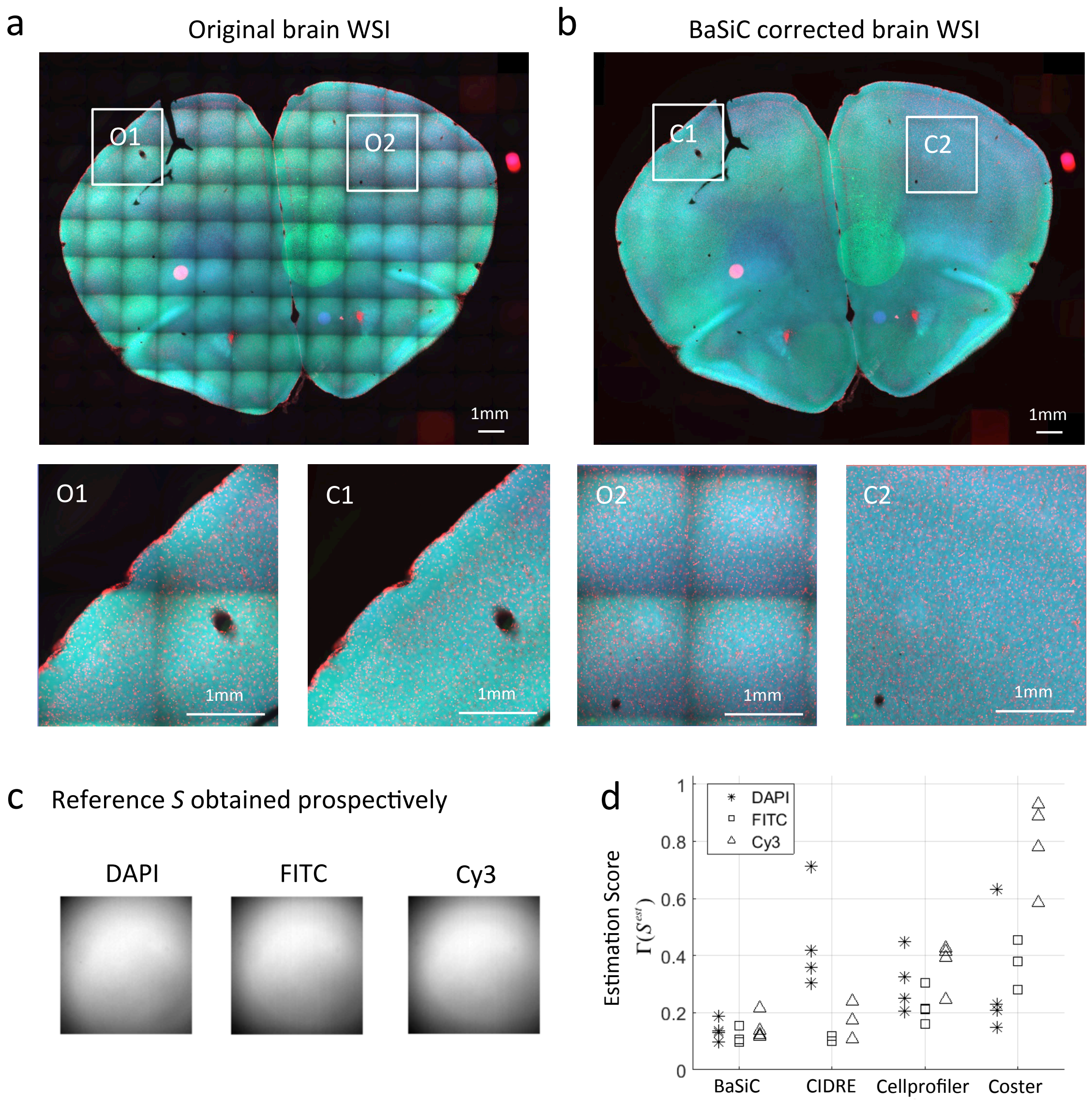

Supplementary Fig. 6: Four WSIs of mouse brain section were collected using three fluorescence channels, DAPI, FTIC and Cy3. For each retrospective method, one estimation was made for each WSI on each channel. Besides, reference dark-field and flat-field were obtained prospectively using a concentrated dye solution. (a) An exemplary original WSI. Intensity inhomogeneities in each image tile give rise to a checkerboard pattern in the stitched WSI. (b) The same WSI after BaSiC correction. With intensity correction, the stitched WSI becomes smooth and seamless. (c) Flat-field S obtained prospectively for all three channels using concentrated dye solution. (d) Estimation score, $\Gamma(S^{est})$, between the estimated S using different retrospective methods and the reference S obtained prospectively. A $\Gamma(S^{est}) = 1$ implies an equivalent error to the baseline error between reference S and a uniform image (no correction). The relatively large error of Coster at Cy3 channel is due to additive stray light and residual excitation light (refer to Supplementary Fig. 4 for details).

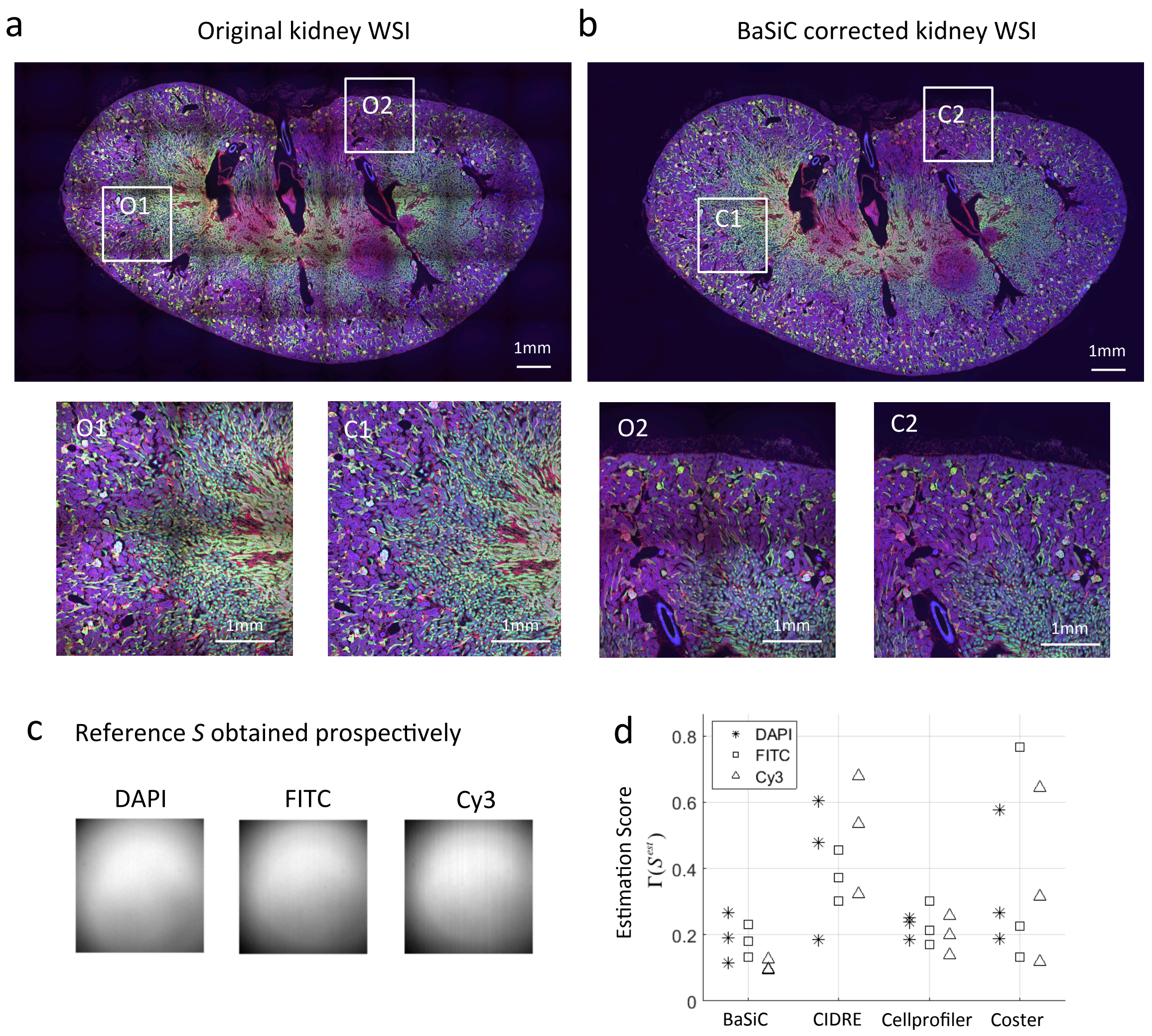

Supplementary Fig. 7: Three WSIs of mouse kidney section were collected using three fluorescence channels, DAPI, FTIC and Cy3. For each retrospective method, one estimation was made for each WSI on each channel. Besides, reference dark-field and flat-field were obtained prospectively using a concentrated dye solution. (a) An exemplary original WSI. Intensity inhomogeneities in each image tile give rise to a checkerboard pattern in the stitched WSI. (b) The same WSI after BaSiC correction. With intensity correction, the stitched WSI becomes smooth and seamless. (c) Flat-field S obtained prospectively for all three channels using concentrated dye solution. (d) Estimation score, $\Gamma(S^{est})$, between the estimated S using different retrospective methods and the reference S obtained prospectively. A $\Gamma(S^{est}) = 1$ implies an equivalent error to the baseline error between reference S and a uniform image (no correction).

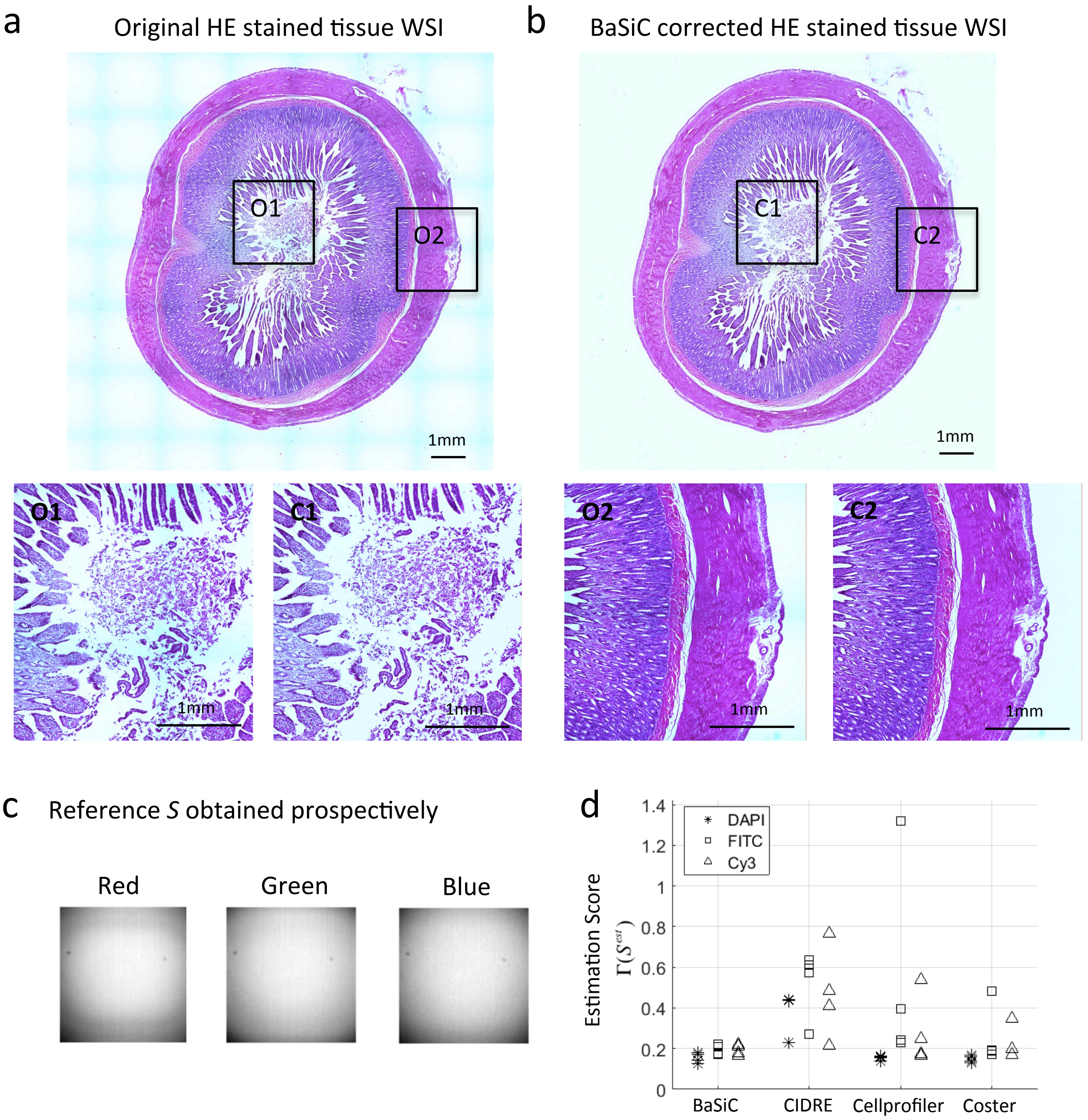

Supplementary Fig. 8: Four WSIs of HE (Hematoxylin and Eosin) stained tissue sections were collected using brightfield microscopy. For each retrospective method, one estimation was made for each WSI on each channel. Besides, reference dark-field and flat-field were obtained prospectively using a concentrated dye solution. (a) An exemplary original WSI. Intensity inhomogeneities in each image tile give rise to a checkerboard pattern in the stitched WSI. (b) The same WSI after BaSiC correction. With intensity correction, the stitched WSI becomes smooth and seamless. (c) Flat-field S obtained prospectively for RGB channels using empty parts of the specimen. (d) Estimation score, $\Gamma(S^{est})$, between the estimated S using different retrospective methods and the reference S obtained prospectively. A $\Gamma(S^{est}) = 1$ implies an equivalent error to the baseline error between reference S and a uniform image (no correction).

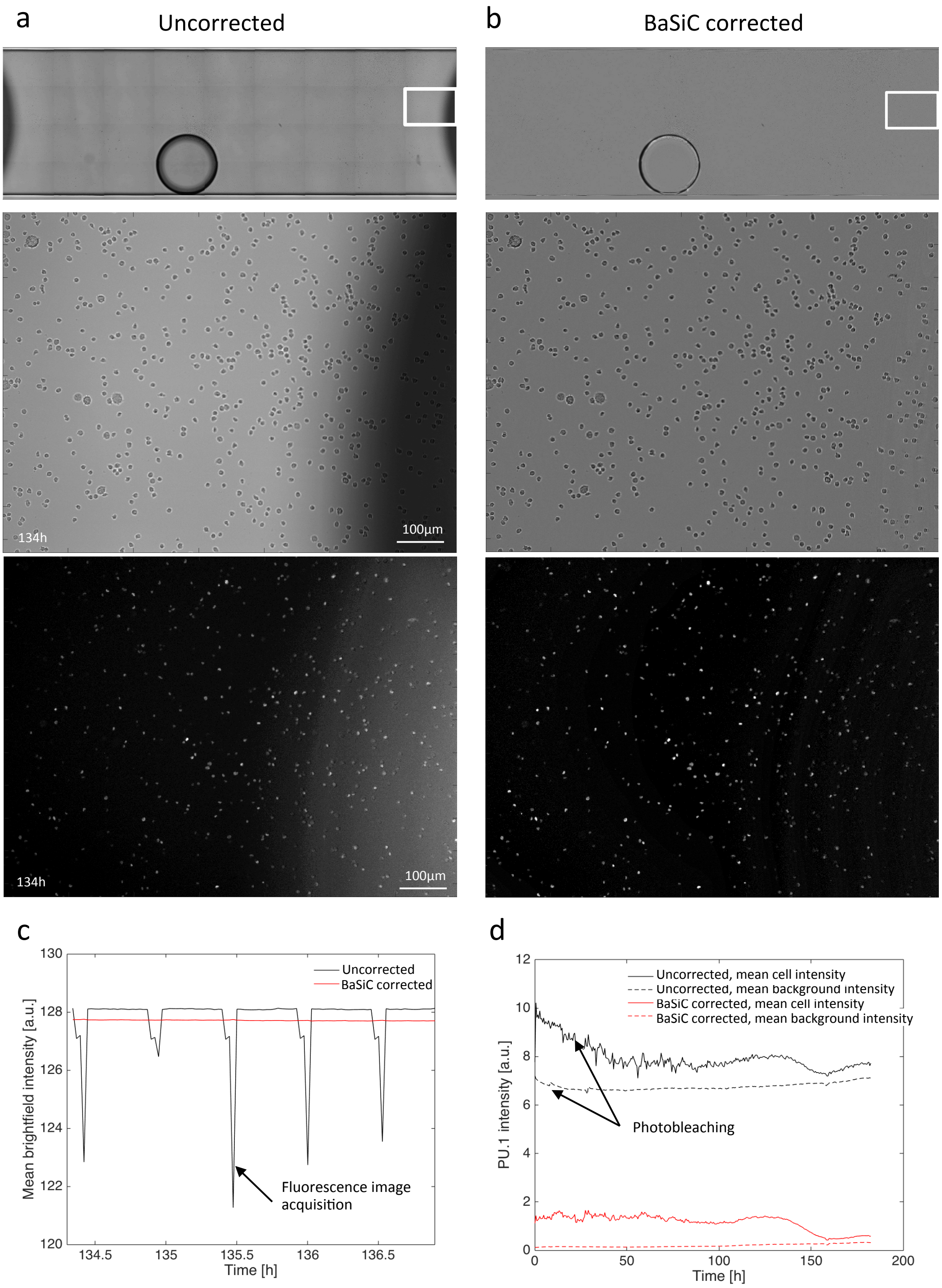

Supplementary Fig. 9: BaSiC corrects both shading in space and baseline drift in time. (a) A culture plate (top) where cells at the culture plate edges are unevenly illuminated, which results in a low brightfield intensity (middle) and a high fluorescence intensity (bottom). (b) BaSiC corrects the shading at the plate edges for both brightfield and fluorescence images. Yet the shading introduced by the disturbing bubble cannot be completely removed, as the bubble changes its size and position over time. (c) Besides shading in space, BaSiC also removes the negative 'spikes' of the mean brightfield intensity at every $20th$ frame, which happens due to sudden switching of microscopy settings to capture fluorescence images. (d) In a similar way, BaSiC resolves the temporal baseline drift of the fluorescence images due to photobleaching.

Supplementary Note 1: Prospective shading correction

For light microscopy, a measured image, $I^{meas}(x)$, can be approximated by a linear model as:

$$I^{meas}(x) = I^{true}(x) * S(x) + D(x) \quad (1)$$

where $I^{true}(x)$ is the shading-free true image, the multiplicative flat-field S represents variation in effective illumination, and the additive dark-field D includes camera offset, thermal noise and possibly stray light and residual excitation light due to incomplete filtering. Although stray light and residual excitation light disappear when the light source is switched off, they have a similar additive effect as the 'true' dark-field components like camera offset and thermal noise and thus cannot be mathematically separated. A prospective approach for shading correction attempts to measure the flat-field S and the dark-field D using special reference images and hence corrects the shading effect by inverting the model.

For bright-field microscopy, the reference images are usually captured at a blank field of view (also known as white reference) taken immediately after each exposure as an approximation of the flat-field S. A median averaging of several white reference images can improve the robustness of approximating S against typical image artefacts such as dusts. Here, the contribution of the dark-field D is usually negligible as the signal I^{true} is usually strong. As a result, many whole slide image scanners incorporate this white reference correction procedure as a built-in function and often output shading-corrected images directly.

For fluorescence microscopy, the calibration process is more demanding, as S depends on optical filters and differs between imaging channels and objectives [1]. Conventional approaches acquire S using specific plastic calibration slides [2], empty glass slides [3] or images of a concentrated fluorescent dye [4, 5]. In practice, correction using plastic calibration slides is often not optimal, due to a different thickness as compared the real specimen (Kurt's Microscopy Blog, `http://nic.ucsf.edu/blog/?p=922`). Moreover, the signal from an empty glass slide can be weak and is easily influenced by the additive D (Supplementary Fig. 4a). In comparison, concentrated dye has a similar thickness as the real specimen and thus gives rise to a more robust approximation of S due to the strong signal of pure dye. A detailed protocol of concentrated dye solution for shading correction is shown in Kurt's Microscopy Blog (`http://nic.ucsf.edu/blog/?p=1032`).

In summary, prospective methods can lead to effective shading correction yet acquiring reference images requires extra effort. This extra effort is often too prohibitive for many bioimaging researchers so that about half of researchers do not use any flat-field correction according to the survey of Smith et al. [6]. Additionally, most of the current protocols cannot measure stray light and residual excitation light in the light path and may result in insufficient correction (Supplementary Fig. 4b).

Supplementary Note 2: Retrospective shading correction

In contrast to a prospective approach, a retrospective approach requires only the actual images acquired during an experiment and can be carried out after image acquisition. It can be used to correct illumination anomalies in absence of calibration or incorrect calibration. Generally, retrospective methods can be categorised into: (i) single-image based and (ii) multi-image based approaches.

Estimation of shading from a single image is difficult but possible in certain circumstances. Most single-image based methods assume the background to be a scaled version of flat-field, S. The focus of this approach is to separate the image background from the foreground via, e.g. rolling ball algorithm which removes the foreground by morphological filtering [7], smoothing with a large gaussian kernel size [8], approximating background using polynomial fitting or spline fitting [9], or separating background/foreground using a clustering method [10]. A bottleneck of these single-image based methods is that their performance often deteriorates as the size and the number of the foreground objects increases [9].

A multiple-image based approach utilises the shared S among multiple images and is usually more reliable. Among the published multi-image correction methods in the literature, the first type of these methods (e.g. [11, 12]) segments the foreground/background for each image and uses interpolation/extrapolation to obtain S and D. The major drawback of this approach is that segmentation is a challenge of its own for many microscopy images and may not be robust to varying densities. The second type takes advantage of statistical properties of an image sequence and hence does not require a segmentation step. Our proposed algorithm, BaSiC, and all methods included in the paper (CIDRE [6], Cellprofiler [13,14] and Coster [15], refer to Online Methods for the details of each method) belong to this second category. Among these methods, only BaSiC and CIDRE can simultaneously estimate S and D, while the other methods either ignore D or require to prospectively obtain D as an input.

Supplementary Note 3: BaSiC

Mathematical model of a measured image sequence

A measured image sequence $I^{meas} = I_1^{meas}, ..., I_n^{meas} \in \mathbb{R}^{p\times q}$, can be related to its uncorrupted correspondence $I^{true} = I_1^{true}, ..., I_n^{true}$ by [6]

$$I_i^{meas}(x) = I_i^{true}(x) * S(x) + D(x), \tag{2}$$

where the multiplicative term $S(x)$ represents the change in effective illumination across an image (also known as flat-field); the additive term, $D(x)$, is dominated by camera offset and thermal noise, which are present even if no light is incident on the sensor (also known as dark-field). We further decompose the shading-free true image, $I_i^{true}(x)$, into the sum of a spatially-constant baseline signal, B_i, and the spatially varying $F_i(x)$, the foreground (fluorescence) signal of biological relevance [15] . Hence the full model for the ith image of a measured image sequence becomes

$$I_i^{meas}(x) = (B_i + F_i(x)) * S(x) + D(x). \tag{3}$$

For time-lapse microscopy data, B_i is usually not constant between frames due to photobleaching of the background medium and may vary between experimental conditions. Hence it is important to correct the temporal drift of B_i to recover the true dynamics of foreground signal, $F_i(x)$.

Estimating the flat-field S by low rank and sparse decomposition

First we assume that the additive dark-field D is negligible and hence the shading model can be simplified into:

$$I_i^{meas}(x) = I_i^{true}(x) * S(x). \tag{4}$$

The BaSiC correction begins by sorting intensity values of the measured image sequence at each pixel location x in an ascending order, termed $I(x) = [I_1^{sort}(x), ..., I_n^{sort}(x)]$. Since $S(x)$ is a positive multiplicative component, the uncorrupted correspondence is also in an ascending order. For an infinite number of images, $n \to \infty$, $I_i^{sort}(x)$ is the quantile function of intensities in the corrupted images I^{meas} at location x. If the distribution of the foreground objects in the image sequence is independent of location x, any ith quantile, $I_i^{sort}(x)$, is proportional to the flat-field $S(x)$. As a result, for an infinite number of images, any simple statistical measure, such as mean, median, or ith quantile, can be used to recover $S(x)$.

Yet in reality, there is always a limited number of images to be corrected and hence the distribution of the foreground objects is no longer independent of location x. We concatenate each sorted image $I_i^{sort}(x)$ into a column vector, I_i^{sort} using vec : $\mathbb{R}^{p\times q} \to \mathbb{R}^{pq\times 1}$ (from now on, we denote the same variable in image space with (x) and as a column vector without (x)) and construct a measurement matrix $I = [I_1^{sort}, ..., I_n^{sort}]$. We further decompose each column of the matrix, I_i, into:

$$I_i = B_i * S + R_i, \tag{5}$$

where B_i is a spatially-constant scalar and R_i is the residual. For $i = 1, ..., n$, B_i is the image-specific baseline. Hence the measurement matrix can be written as

$$I = I^B + I^R \in \mathbb{R}^{pq\times n}, \tag{6}$$

where the first component, $I^B = [B_1 * S, \ldots, B_n * S] = B \odot S \in \mathbb{R}^{1\times n}$ ($\odot$ denotes column-wise multiplication), is a rank 1 matrix as B_i is a scalar. The second component, I^R, is the residual

matrix which is assumed to be *sparse*, i.e. the residual $I_i^R(x)$ generally occupies only a small fraction of image pixels. Furthermore, it is also reasonable to assume the flat-field, S, to be smooth. Smooth functions are typically *sparse in the Fourier transformed domain.* Hence, we have the following constraint optimisation problem:

$$\begin{aligned} &\min_{B,S} \|I^R\|_0 + \lambda_s \|\mathcal{F}(S(x))\|_0, \\ &\text{subject to } I = I^B + I^R; I^B = B \odot S. \end{aligned} \tag{7}$$

Here $\|\cdot\|_0$ denotes the L_0-norm (i.e. the number of non-zero matrix elements), $\mathcal{F}$ represents the Fourier transform, and λ_s is a weighting parameter, which balances the sparse penalty of two matrices, one in image domain and one in the Fourier transformed domain. The choice of λ_s will be discussed in Supplementary Note 4.

Iterative optimisation of Eq. (7): The optimisation of a L_0-norm is NP-hard [16]. A common trick is to replace the L_0-norm with a L_1-norm. A key difference is the dependence on the magnitude: large coefficients are penalised more heavily in the L_1-norm than small coefficients, whilst the L_0-norm gives the same penalisation regardless of the magnitude. To address this imbalance, an iterative reweighted L_1-norm minimisation was proposed to equalise penalisation of nonzero coefficients [17]. It has been shown that the iterative reweighted L_1-norm has a similar performance as the normal L_1-norm when the matrix has a small number of non-zero coefficients (high sparsity) yet the reweighed L_1-norm is more robust as the number of non-zeros increases (less sparsity). In our case, $\mathcal{F}(S(x))$ has a high sparsity, as a flat-field is usually smooth with most of its energy concentrated in low-frequency bands. By contrast, the sparsity of the residual matrix I^R is less predictable as it is largely dependent on the image number and content (such as the cell density). An increasing number of images leads to an increasing sparsity, e.g., I^R becomes a zero matrix when infinite images are available. Therefore, in order to be robust to a small number of images, we use a reweighted L_1-norm to replace the L_0- norm minimisation of R,

$$\begin{aligned} &\min_{B,S} \|W \circ I^R\|_1 + \lambda_s \|\mathcal{F}(S(x))\|_1 \\ &\text{subject to } I = I^B + I^R; I^B = B \odot S, \end{aligned} \tag{8}$$

where $W \in \mathbb{R}^{pq*n}$ is the weighting matrix and $\circ$ represents the element-wise Hadamard product. The elements of W are initially set to be 1 on iteration 0, and are updated by

$$W^{(k+1)} = \frac{1}{|I^{R,(k)}/I^{B,(k)}| + \epsilon}, \tag{9}$$

where the parameter $\epsilon > 0$ is introduced to provide stability. In each iteration, we simultaneously minimise the L_1-norm of two matrices, in the image and Fourier spaces, respectively. We utilise the Linearized augmented Lagrangian method (LADM) [18], which has also been used in robust PCA [19] and RASL [20]:

$$\begin{aligned} L(S, I^B, I^R, Y, \mu) = &\|W \circ I^R\|_1 + \lambda_s \|\mathcal{F}(S(x))\|_1 \\ &+ \langle Y, I - I^B - I^R \rangle + \mu \|I - I^B - I^R\|_F \end{aligned} \tag{10}$$

Here, Y denotes the Lagrange multiplier, $\langle \cdot, \cdot \rangle$ is the inner product, $\|\cdot\|_F$ is the Frobenius norm and $\mu > 0$ is a penalty parameter. The detailed LADM updating scheme is described in Tab. 1.

Input: Sorted intensity matrix of observations, $I \in \mathbb{R}^{pq\times n}$; setting W according to Eq. (9) using I^B and I^R from last iteration
1: $Y^{(0)} = 0; S^{(0)} = 0; B^{(0)} = 0; R^{(0)} = 0; \mu^{(0)} > 0; \rho > 1; k = 0$
2: **while** not converged **do**
3: $\quad S_F^{(k)} = \mathcal{F}(S(x)^{(k)}); I^{B,(k)} = B^{(k)} \odot S^{(k)};$
4: $\quad S_F^{(k+1)} = \mathcal{T}_{\lambda_s/\mu^{(k)}}\{S_F^{(k)} + \mathcal{F}[\text{avg}_{row}(I - I^{B,(k)} - I^{R,(k)} + Y^{(k)}/\mu^{(k)})]\};$
5: $\quad S^{(k+1)} = \mathcal{F}^{-1}(S_F(x)^{(k+1)}); I^{B,(k+1/2)} = B^{(k)} \odot S^{(k+1)};$
6: $\quad I^{R,(k+1)} = \mathcal{T}_{W/\mu^{(k)}}\{I - I^{B,(k+1/2)} + Y^{(k)}/\mu^{(k)}\};$
7: $\quad B^{(k+1)} = \text{avg}_{col}(I - I^{R,(k+1)}) / \text{avg}_{mat}(I - I^{R,(k+1)}); I^{B,(k+1)} = B^{(k+1)} \odot S^{(k+1)}$
8: $\quad Y^{(k+1)} = Y^{(k)} + \mu^{(k)}(I - I^{B,(k+1)} - I^{R,(k+1)}); \mu^{(k+1)} = \rho\mu^{(k)}; k = k + 1.$
9: **end while**
Output: $(S^{(k)}, I^{B,(k)}, I^{R,(k)})$; $S^{(k)}$ is the estimated unnormalised flat-field. A normalised flat-field can be obtained by dividing $S^{(k)}$ using its mean value.
Note: $\text{avg}_{row}, \text{avg}_{col}, \text{avg}_{mat}$ are row, column and matrix mean respectively; $\mathcal{T}_\varepsilon(\theta) = \text{sgn}(\theta)\max(|\theta| - \varepsilon, 0)$ is the scalar shrinkage operator.

Table 1: LADM updating scheme for optimisation of Eq. (7)

Simultaneous estimation of the flat-field S and the dark-field D

Considering the additive component D, we replace Eq. (5) with:

$$I_i = B_i * S + D + I_i^R. \tag{11}$$

It is obvious that the above equation does not have a unique mathematical solution (suppose D^* is one solution, then $D^* - z * S, z \in \mathbb{R}$ is another solution). As a result, we decompose D as a sum of its mean D^Z and the residual D^R, yielding:

$$\begin{aligned} I_i &= B_i * S + D^Z + D^R + I_i^R \\ &= (D^Z + B_i) * S + D^Z(1 - S) + D^R + I_i^R \\ &= (D^Z + B_i) * S + Z + I_i^R \end{aligned} \tag{12}$$

The sum of the first two term of Eq. (12) forms the low rank component (rank<=2), as $D^Z + B_i$ is a scalar; the last term, I_i^R, is the sparse term. Without any regularization on D and Z, it would be difficult to obtain a stable estimation. We assume D to be nearly flat and regulate the L_1 norm of D^R in both image and Fourier domains, to ensure that the residual D^R is small and smooth. Another regularization on D is that D^Z should be limited by $[0, \min(I)]$. Adding the regularization on D and Z, our minimization problem turns into

$$\begin{aligned} &\min_{B,S,D} \|W \circ I^R\|_1 + \lambda_s\|\mathcal{F}(S(x))\|_1 + \lambda_d\|\mathcal{F}(D^R(x))\|_1 + \lambda_d\|D^R\|_1 \\ &\text{subject to } I = I^B + I^R; I^B = B \odot S \oplus Z; \\ &\qquad Z = D^Z(1 - S) + D^R; D^Z \in [0, \min(I)]. \end{aligned} \tag{13}$$

where $\oplus Z$ denotes a column-wise addition. The minimization of the first two terms of Eq. (13) is similar to the LADM updating of Eq. (8), with $I^B = B \odot S$ replaced by $I^B = B \odot S \oplus Z$ (Z is also initialized with zeros). Yet the conditional minimization of the last two terms is not straightforward. Our proposed strategy is to first calculate D^Z by least square fitting and then optimize D^R using the shrinkage operator (refer to Tab. 2 for a detailed updating scheme).

**Input**: Sorted intensity matrix of observations, $I \in \mathbb{R}^{pq\times n}$; $B^{(k)} \in \mathbb{R}^{1\times n}$; $S^{(k)} \in \mathbb{R}^{p\times q}$; $I^{R,(k)} \in \mathbb{R}^{pq\times n}$ from Tab. 1
1: Calculate D^Z using least square fitting:
2: divide the image into two regions $S^{(k)} >= 1$ and $S^{(k)} < 1$
3: $A = \left(\text{avg}_{S^{(k)}>=1}(I - I^{R,(k)}) - \text{avg}_{S^{(k)}<1}(I - I^{R,(k)})\right) / \text{avg}_{mat}(I - I^{R,(k)})$
4: $D^Z = \left(\sum_i B_i^2 \sum_i A_i - \sum_i B_i \sum_i A_i B_i\right) / \left(\sum_i A_i \sum_i B_i - n \sum A_i B_i\right)$
6: $D^Z = \min(D^Z, 0)$; $D^Z = \max(D^Z, \min(I(:,1)))$;
7: Optimize D^R:
8: $Z = \text{avg}_{col}(I - I^{R,(k)}) - \text{avg}_{col}(B^{(k)} \odot S^{(k)})$;
9: $D^R = Z - \text{avg}_{col}(Z) - D^Z * (\text{avg}_{col}(S^{(k)}) - S^{(k)})$;
10: $D^R = \mathcal{T}_{\lambda_d/\mu^{(k)}} D^R$; $D^R = \mathcal{F}^{-1}(\mathcal{T}_{\lambda_d/\mu^{(k)}} \mathcal{F}(D^R(x)))$;
11: $Z^{(k)} = D_z * (\text{avg}_{col}(S^{(k)}) - S^{(k)}) + D^R$; $D^{(k)} = D^Z + D^R$.
**Output**: $D^{(k)} \in \mathbb{R}^{p\times q}$, dark-field.
**Note**: $\text{avg}_{row}, \text{avg}_{col}, \text{avg}_{mat}$ are row, column and matrix mean respectively; $\mathcal{T}_\varepsilon(\theta) = \text{sgn}(\theta)\max(\lvert\theta\rvert - \varepsilon, 0)$ is the scalar shrinkage operator.

Table 2: Updating of the dark-field D of Eq. (13) in one iteration

Estimation of the per-image baseline B_i

After sorting intensities, the estimated B_i alongside S and D is not the baseline of the original images, but that of the sorted images. A straightforward approach to obtain B_i for the original images is to construct the measurement matrix as $I = [I_1^{sort}, ..., I_n^{sort}, I_1^{meas}, ..., I_n^{meas}]$, hence the second part of the obtained B_i corresponds to the desired baseline.

However, the computational complexity to decompose the new measurement matrix will be twice of the original matrix that is composed of sorted images only, as the new matrix has twice the number of columns as the original one. This is particularly problematic for background correction of long-term time-lapse movies with thousands of frames, in which B_i has to be computed for every frame. Therefore, we propose a two-step strategy to estimate B_i: the first step is to compute S and D only, using the matrix with sorted intensities; in the second step, we estimate B_i using the unsorted measurement matrix as $I = [I_1^{meas}, ..., I_n^{meas}]$ and the estimated S and D from step 1 as model inputs. Hence, the optimisation problem becomes:

$$\begin{aligned} &\min_B \|W \circ I^R\|_1 \\ &\text{subject to } I = I^B + I^R; I^B = B \odot S^* \oplus D^*, \end{aligned} \tag{14}$$

where S^* and D^* are the solutions of Eq. (13) using sorted images. In comparison to Eq. (13), solving Eq. (14) numerically is much faster, due to a reduced degree of freedom. For an image sequence with a large number of frames, we can accelerate the estimation of S and D in the first step by sampling a representative image subset (Fig. 2 shows that BaSiC needs at most a few hundred images to achieve a reliable estimation). The estimation of B_i in the second step with Eq. (14) is fast and can be further accelerated by parallel computing, e.g., we can divide a long image sequence into several short sequences and compute B_i for each short sequence in parallel. This implementation optimises both computational time and memory consumption.

Supplementary Note 4: Automatic parameter settings

The parameters λ_s and λ_d in Eq. (13) control the strength of regularisation on the flat-field S and the dark-field D, respectively. Large λ values will increase the smoothness/flatness of the estimated flat-field/dark-field, whilst lower values will allow them to fit the data more freely.

In general, the regularisation terms become less necessary as more images are provided, e.g., when an infinite number of images is available, simply taking the per-pixel mean of these images will be a good estimation of S. Inspired by this, we are able to build a strategy to automatically determine λ values based on the smoothness of the per-pixel mean of all images: if the mean is already smooth, which is usually the case when a large number of images available, we set λ values to be small and vice verse:

$$\lambda_s = \frac{\|\mathcal{F}(\bar{I}_{norm})\|_1}{800}, \quad \lambda_d = \frac{\|\mathcal{F}(\bar{I}_{norm})\|_1}{2000}, \tag{15}$$

where $\bar{I}_{norm}$ is the normalised per-pixel mean of all images. The denominators, 800 and 2000, are empirically determined using synthetic data, where we try to minimise the errors between our repeatedly estimated S and D from bootstrapped samples and the ground-truth S and D (generation of synthetic images of different cell densities is described in Supplementary Note 6). In practice, we find that the performance of BaSiC is robust to a wide range of denominators and the error is minimal for the chosen numbers. In all our experiments presented, we use this automatic parameter setting strategy, which is demonstrated to work well for a wide range of different image types. Yet we provide the possibility to manually determine parameters and overwrite the default setting in the BaSiC plugin.

The number of reweighing iterations of sparse coefficients is also dynamically adjusted: the reweighing terminates when the difference between the estimations of two consecutive iterations is smaller than a predetermined threshold (we set to be 0.1%) or the iteration number exceeds the maximum number of iterations (10 in our setting).

Supplementary Note 5: Connection to statistics

In this section we show that under certain conditions, the solution of our optimisation is mathematically equivalent to taking the per-pixel mode of all images, thus making a connection between our method and statistical theory.

In Eq. (7), if we neglect the smooth regularisation by setting $\lambda_s = 0$, we get:

$$\min_{B_i, S(x)} \sum_{i,x} \|I_i(x) - B_i * S(x)\|_0, \tag{16}$$

in which $\| * \|_0$ is zero only when $I_i(x) = B_i * S(x)$. The maximal equivalence is achieved by taking the mode:

$$S(x) = \operatorname*{mode}_i \left(\frac{I_i(x)}{B_i} \right), \quad B_i = \operatorname*{mode}_x \left(\frac{I_i(x)}{S(x)} \right). \tag{17}$$

In our optimisation we use an iterative reweighed L_1-norm minimisation to replace a direct L_0-norm minimisation as the later is usually NP hard [16]. Compared to the common unweighted L_1 relaxation, the reweighed L_1 algorithm can improve the recovery of sparse signals as it actually minimises a log-sum penalty function [17]:

$$\min_{x \in \mathbb{R}} \sum_x \log\left(|x| + \epsilon\right). \tag{18}$$

The above problem can be minimised using a gradient decent algorithm in an iterative weight updating fashion:

$$\frac{\partial f}{\partial |x|} = \frac{1}{|x| + \epsilon}, \tag{19}$$

Among three potential penalty functions: $f_0(t) = 1_{(t \neq 0)}, f_1(t) = |t|, f_{\log,\epsilon} \propto \log(1 + |t|/\epsilon)$, the L_0 penalty function f_0 has an infinite slope at $t = 0$, while its convex L_1 relaxation f_1 has a unit slope. The log penalty function $f_{\log,\epsilon}$ tends to f_0 as $\epsilon \to 0$ and to f_1 as $\epsilon \to \infty$. In principle, ϵ should be set as small as possible to obtain a close resemblance to the L_0 norm. Yet $f_{\log,\epsilon}$ is a concave function and as $\epsilon \to 0$, it becomes more likely that the iterative reweighed algorithm will get stuck in an undesirable local minimum. In BaSiC, we set $\epsilon = 0.1$, which provides a smooth gradient descent while still improving upon the unweighted L_1 algorithm for sparse recovery.

Supplementary Note 6: Generation of synthetic data

Synthetic images as used in Supplementary Fig. 2 are generated using the following procedure:

1. We generate perfect shading-free images using the fluorescence simulation tool in Lehmussola et al. [21]. Each image is of size 400×400 pixels, with three different settings: (a) 40 cells, fluorescently labeled nuclei; (b) 40 cells, fluorescently labeled cytoplasm; c) 160 cells, fluorescently labeled cytoplasm. The mean cell density (i.e. the proportion of the area occupied by the cell foreground) of the three image types are 7.3%, 35.8% and 78.6%, respectively.

2. We simulate the shading effect by corrupting the shading-free images with a preset multiplicative flat-field $S(x)$ and an additive dark-field $D(x)$. In order to make our simulation as realistic as possible, we use prospectively obtained $S(x)$ and $D(x)$ using a concentrated dye solution as our shading profiles.

Supplementary Note 7: Software usage, practical tips and precautions

The BaSiC plugin[1] can be installed to Fiji/ImageJ following the instruction given in the **readme** file in the software folder. We present five exemplary applications to explain the software usage:

1. BaSiC is used to correct shading in a brain WSI and hence allows seamless stitching.
2. BaSiC is used to correct shading in a cell culture dataset and hence improves contrast at the image corners where the illumination is weak.
3. BaSiC is used to correct both shading and temporal flashing (due to varying experimental conditions) of the brightfield channel of a time-lapse movie of differentiating mouse hematopoietic stem cells.
4. BaSiC is used to correct both shading and temporal drift (due to photobleaching in the background medium) of the fluorescence channel of the time-lapse movie of differentiating mouse hematopoietic stem cells.
5. BaSiC is used to correct both shading and temporal drift (due to photobleaching in the background medium) of the fluorescence channel of the time-lapse movie of differentiating mouse embryonic stem cells. Here the correlation between frames is high due to slow movement of cells, which challenges the basic assumption of BaSiC (see below for explanation). Yet we can tune smooth regularisation parameters to adapt BaSiC's performance.

Except for the last application, the first four demo applications use automatic parameter setting in BaSiC and hence no manual parameter tuning is required.

In practice, the estimation of the dark-field D is not always necessary. For example, in bright-field images, the contribution of D is negligible as the signal is strong. Besides, when raw images (typically in 16-bit or 32-bit format) are converted into 8-bit by scaling from min-max to 0-255, D might be already corrected during the rescaling. In such situations, we provide an option in BaSiC to neglect D. Estimating the flat-field S alone is computationally faster and more stable than the simultaneous estimation of both flat-field and dark-field.

In general, BaSiC's computation is fast: it usually processes hundreds of images within minutes at a standard laptop. A key factor for such a fast computation is that we always rescale the input images to 128x128 pixels. This resizing saves computational expense without losing accuracy since S and D are usually smooth and B_i is a scalar. However, the computational complexity of BaSiC linearly increases with the number of images, as the number of columns of the matrix I (Eq. (13)) to be decomposed is equivalent to the number of images.

For an image sequence with a large number of images, we can accelerate the estimation of S and D by sampling a representative image subset. The required number of images for BaSiC depends on many factors, such as confluency, thickness of background medium and uniformity of foreground fluorescence. The required image number is smallest for cells of low confluency and a thick medium (such as for MICROFLUID dataset in Supplementary Fig. 3). For images without any background signal (such as confocal images or cells without medium, e.g. HIST-CONFOCAL and HCS-ACTIN, Supplementary Fig. 3), estimation has

[1] The BaSiC software, data and usage of all exemplary applications can be downloaded from `https://www.helmholtz-muenchen.de/icb/research/groups/quantitative-single-cell-dynamics/software/basic/index.html`.

to rely on the foreground alone, and hence uniformity of foreground fluorescence will play an important role, i.e., a more uniform foreground requires fewer images. Nevertheless, as shown in Supplementary Fig. 3, even for challenging datasets with no medium and varying foreground, BaSiC requires at most a few hundred images to reach a reliable estimation.

BaSiC also provides an option to separate shading correction (estimation of S and D) and background correction (estimation of B_i). This provides a very effective strategy to process long-term time-lapse movies with thousands of frames. First, we can compute S and D using a small image subset, which is much faster than using the whole image sequence. In the second step we use the precomputed S and D as inputs and compute per-image B_i in a block-wise fashion, i.e., we divide a long image sequence into several short sequences and compute B_i for each short sequence either sequentially or in parallel. Hence, this implementation optimises both computational time and memory consumption.

Precautions

As any shading correction method, BaSiC also has limitations. One key assumption of BaSiC and all other previously mentioned retrospective methods is that the foreground of every image to be processed should be uncorrelated with the foreground of every other image. This assumption can be violated for time-lapse movies of static and quasi-static objects, e.g. a single cell of high magnification that is always in the centre of the field of view. In this case, BaSiC would consider the consistently higher image intensities in the centre of the field of view as a local increase in S, causing removal of the true fluorescence variability. Nevertheless, in practice, BaSiC still has some tolerance to correlation, e.g., it performs well in a movie of proliferating and slowly moving embryonic stem cell colonies (as shown in Supplementary Video 2), in which consecutive frames are correlated. Meanwhile, the regularisation λ_s can be used to tune the resulting model so that it is more suitable for correlated images. Larger value of λ_s leads to a smoother estimate of the low rank component, thus rejecting small static objects in the estimated S. Another useful strategy is to take samples with a large time gap in between to make images less correlated. Nevertheless, we suggest users to visually inspect the estimated shading profiles before make a correction in these challenging cases: a smooth S usually indicates good shading correction, while local inhomogeneity that comes from highly corrected foreground objects is a hint of non-optimal correction.

It should also be noted that BaSiC can compensate background variation, no matter if it is caused by bleaching or by switching microscopy settings, only when the foreground fraction of the image is lower than 50% (otherwise B_i converges to the mean foreground intensity). This does not affect the practical usage of BaSiC as photobleaching happens mostly at the beginning of a time-lapse movie where the cell density is sparse. In contrast, when BaSiC is applied to only correct shading effects for static images, the requirement of sparse cell density is not necessary as it is robust to different levels of cell density.

Supplementary References

[1] van den Doel, L. R. *et al.* Quantitative evaluation of light microscopes based on image processing techniques. *Bioimaging* **6**, 138–149 (1998).

[2] Varga, V. S. *et al.* Scanning fluorescent microscopy is an alternative for quantitative fluorescent cell analysis. *Cytometry. Part A : the journal of the International Society for Analytical Cytology* **60**, 53–62 (2004).

[3] Young, I. T. Shading correction: compensation for illumination and sensor inhomogeneities. *Current protocols in cytometry* **Chapter 2**, Unit 2.11 (2001).

[4] Model, M. A. & Burkhardt, J. K. A standard for calibration and shading correction of a fluorescence microscope. *Cytometry* **44**, 309–16 (2001).

[5] Model, M. A. Intensity calibration and flat-field correction for fluorescence microscopes. *Current protocols in cytometry* **68**, 10.14.1–10.14.10 (2014).

[6] Smith, K. *et al.* CIDRE: an illumination-correction method for optical microscopy. *Nature methods* **12**, 404–406 (2015).

[7] Sternberg, S. R. Biomedical Image Processing. *Computer* **16**, 22–34 (1983).

[8] Leong, F. J. W.-M., Brady, M. & McGee, J. O. Correction of uneven illumination (vignetting) in digital microscopy images. *Journal of clinical pathology* **56**, 619–21 (2003).

[9] Likar, B., Maintz, J. B., Viergever, M. a. & Pernus, F. Retrospective shading correction based on entropy minimization. *Journal of microscopy* **197**, 285–95 (2000).

[10] Schwarzfischer, M. *et al.* Efficient fluorescence image normalization for time lapse movies. In *Proc. Microscopic Image Analysis with Applications in Biology* (2011).

[11] Piccinini, F., Lucarelli, E., Gherardi, A. & Bevilacqua, A. Multi-image based method to correct vignetting effect in light microscopy images. *Journal of microscopy* **248**, 6–22 (2012).

[12] Kask, P., Palo, K., Hinnah, C. & Pommerencke, T. Flat field correction for high-throughput imaging of fluorescent samples. *Journal of microscopy* (2016).

[13] Shariff, A., Kangas, J., Coelho, L. P., Quinn, S. & Murphy, R. F. Automated image analysis for high-content screening and analysis. *Journal of biomolecular screening* **15**, 726–34 (2010).

[14] Singh, S., Bray, M.-A., Jones, T. R. & Carpenter, A. E. Pipeline for illumination correction of images for high-throughput microscopy. *Journal of microscopy* **256**, 231–6 (2014).

[15] Coster, A. D., Wichaidit, C., Rajaram, S., Altschuler, S. J. & Wu, L. F. A simple image correction method for high-throughput microscopy. *Nature methods* **11**, 602 (2014).

[16] Foucart, S. & Rauhut, H. *A Mathematical Introduction to Compressive Sensing* (Birkhaeuser, 2013).

[17] Candes, E., Wakin, M. & Boyd, S. Enhancing Sparsity by Reweighted L1 Minimization. *Journal of Fourier Analysis Application* (2008).

[18] Lin, Z., Liu, R. & Su, Z. Linearized alternating direction method with adaptive penalty for low-rank representation. In Shawe-taylor, J., Zemel, R., Bartlett, P., Pereira, F. & Weinberger, K. (eds.) *Advances in Neural Information Processing Systems 24*, 612–620 (2011).

[19] Candès, E., Li, X., Ma, Y. & Wright, J. Robust Principal Component Analysis ? *Journal of the ACM (JACM)* (2011).

[20] Peng, Y., Ganesh, A., Wright, J., Xu, W. & Ma, Y. RASL: robust alignment by sparse and low-rank decomposition for linearly correlated images. *IEEE transactions on pattern analysis and machine intelligence* **34**, 2233–46 (2012).

[21] Lehmussola, A., Ruusuvuori, P., Selinummi, J., Huttunen, H. & Yli-Harja, O. Computational framework for simulating fluorescence microscope images with cell populations. *IEEE transactions on medical imaging* **26**, 1010–6 (2007).
